# Intraoperative Assessment of Anatomical Resection Margin Using a Novel Asymmetric Linear Stapler: An Initial Single Institution Experience

**DOI:** 10.1093/icvts/ivag140

**Published:** 2026-05-09

**Authors:** Yooyoung Chong, Kyung-Hee Kim, Hyun Jin Cho, Calvin S.H Ng, Sandeep J Khandhar, Marion Durand, Toshihiko Sato, Mingyon Mun, Min-Woong Kang

**Affiliations:** Department of Thoracic and Cardiovascular Surgery, Chungnam National University Hospital, Chungnam National University School of Medicine, Daejeon 35015, Republic of Korea; Department of Pathology, Chungnam National University Hospital, Chungnam National University School of Medicine, Daejeon 35015, Republic of Korea; Department of Thoracic and Cardiovascular Surgery, Chungnam National University Hospital, Chungnam National University School of Medicine, Daejeon 35015, Republic of Korea; Department of Surgery, Prince of Wales Hospital, The Chinese University of Hong Kong, Shatin, N.T., Hong Kong, China; Division of Thoracic Surgery, Virginia Cancer Specialists, Inova Fairfax Hospital, Fairfax, VA 22031, United States; Thoracic Surgery Department, Groupe Hospitalier Privé Ambroise Paré Hartmann, Neuilly-Sur-Seine 92200, France; Department of General Thoracic, Breast and Pediatric Surgery, Fukuoka University School of Medicine and Hospital, 7-45-1 Nanakuma, Jonan-Ku, Fukuoka City, Fukuoka, 814-0180, Japan; Department of Thoracic Surgical Oncology, Cancer Institute Hospital, Japanese Foundation for Cancer Research, Tokyo 135-8550, Japan; Department of Thoracic and Cardiovascular Surgery, Chungnam National University Hospital, Chungnam National University School of Medicine, Daejeon 35015, Republic of Korea

**Keywords:** lung cancer, sublobar resection, resection margin, linear stapler, pathology

## Abstract

**Objectives:**

Intraoperative assessment of resection margins during pulmonary resection is limited by tissue compression and distortion caused by conventional staplers, which hinder direct pathological evaluation of the anatomical resection margin. We report our initial clinical experience with a novel asymmetric linear stapler (NALS) designed to provide direct access to the specimen-side anatomical resection margin for intraoperative histologic assessment.

**Methods:**

We retrospectively reviewed patients who underwent pulmonary resection using the NALS between July 2024 and December 2025. Intraoperative frozen-section examination was performed for parenchymal and/or bronchial margins when malignancy was suspected or confirmed. Margin status, margin distance, and frozen–permanent pathological concordance were analysed descriptively.

**Results:**

Among 226 patients who underwent pulmonary resection using the NALS, 206 resection margins (144 parenchymal and 62 bronchial) were evaluated. Intraoperative frozen-section examination identified margin positivity in 12 margins (5.8%). Residual tumour was confirmed on permanent pathology in 4 of 8 parenchymal frozen-positive margins, whereas all bronchial frozen-positive margins showed benign or reactive changes. No false-negative frozen-section findings were identified. Among negative margins with available measurements, 66.7% of parenchymal margins and 84.5% of bronchial margins measured at least 10 mm.

**Conclusions:**

In this initial experience, intraoperative margin assessment using the NALS was feasible and enabled structured evaluation of the anatomical resection margin during pulmonary resection. Because no control group using conventional staplers was included, these findings should be interpreted as a feasibility assessment rather than evidence of superiority over standard stapling devices. Further multicentre studies with controlled comparisons and long-term oncologic follow-up are warranted.

## Graphical abstract

**Figure:**
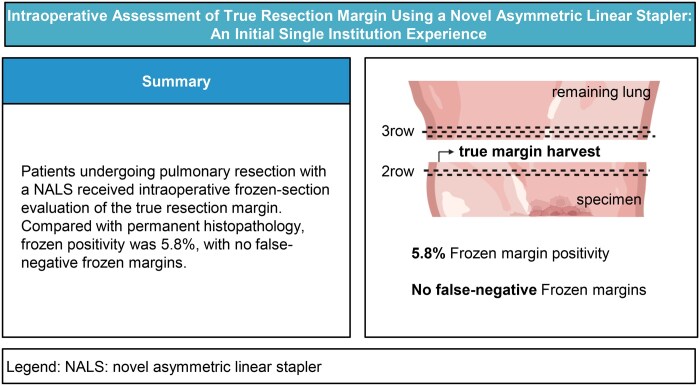

## INTRODUCTION

Complete tumour removal with negative resection margins remains a fundamental principle of curative pulmonary resection for lung cancer.^1–3^ With the widespread adoption of video-assisted thoracoscopic surgery (VATS), linear staplers have become integral to minimally invasive pulmonary resection because they reduce surgical trauma, operative time, postoperative air leak and blood loss.^4–6^ However, conventional stapling systems also impose intrinsic limitations on oncologic margin assessment by causing tissue compression and deformation at the resection plane.^6–9^

Accurate intraoperative margin evaluation is particularly important in pulmonary resection because margin adequacy directly affects oncologic completeness and may influence immediate surgical decision-making.^10–13^ In routine practice, pathological assessment after stapled pulmonary resection is commonly performed on the inner edge of the staple line rather than on the anatomical resection margin located at the outer edge of the stapled specimen.^8^^,^^14^ Because the adjacent lung parenchyma is mechanically compressed and distorted and staples are rarely removed for detailed histopathologic examination, the assessed margin may represent a surrogate rather than the true anatomical resection plane.^6^^,^^8^ As a result, reported margin distance may not accurately reflect the actual intraoperative margin achieved, and frozen-section assessment remains vulnerable to sampling error and interpretive uncertainty.^8^^,^^11^^,^^12^^,^^15–19^

This issue has become increasingly relevant with the expanding role of parenchyma-sparing surgery. Randomized trials have shown that, in selected patients with early-stage lung cancer, sublobar resection can achieve oncologic outcomes compared to lobectomy.^20–23^ In this setting, reliable intraoperative margin assessment is particularly important because limited resections inherently reduce the margin for error.^11^^,^^12^^,^^17^ Recent evidence has shown that intraoperative frozen-section assessment can identify compromised margins and permit immediate additional resection, thereby reducing the incidence of incomplete resection.^24^^,^^25^ Despite this clinical need, currently available linear staplers are not specifically designed to facilitate direct pathological assessment of the anatomical resection margin.^6–8^

To address this gap, a novel asymmetrical linear stapler (NALS) has been developed and previously described in preclinical and technical studies.^9^^,^^26–28^ Its asymmetric staple-line configuration allows targeted access to the anatomical resection margin on the specimen side, thereby facilitating direct intraoperative pathological assessment of the actual resection plane. Accordingly, the present study reports our initial single-institution clinical experience with this device, with a focus on the feasibility of structured intraoperative anatomical margin assessment during pulmonary resection.

## MATERIALS AND METHODS

### Study design and patient selection

**Figure 1.**
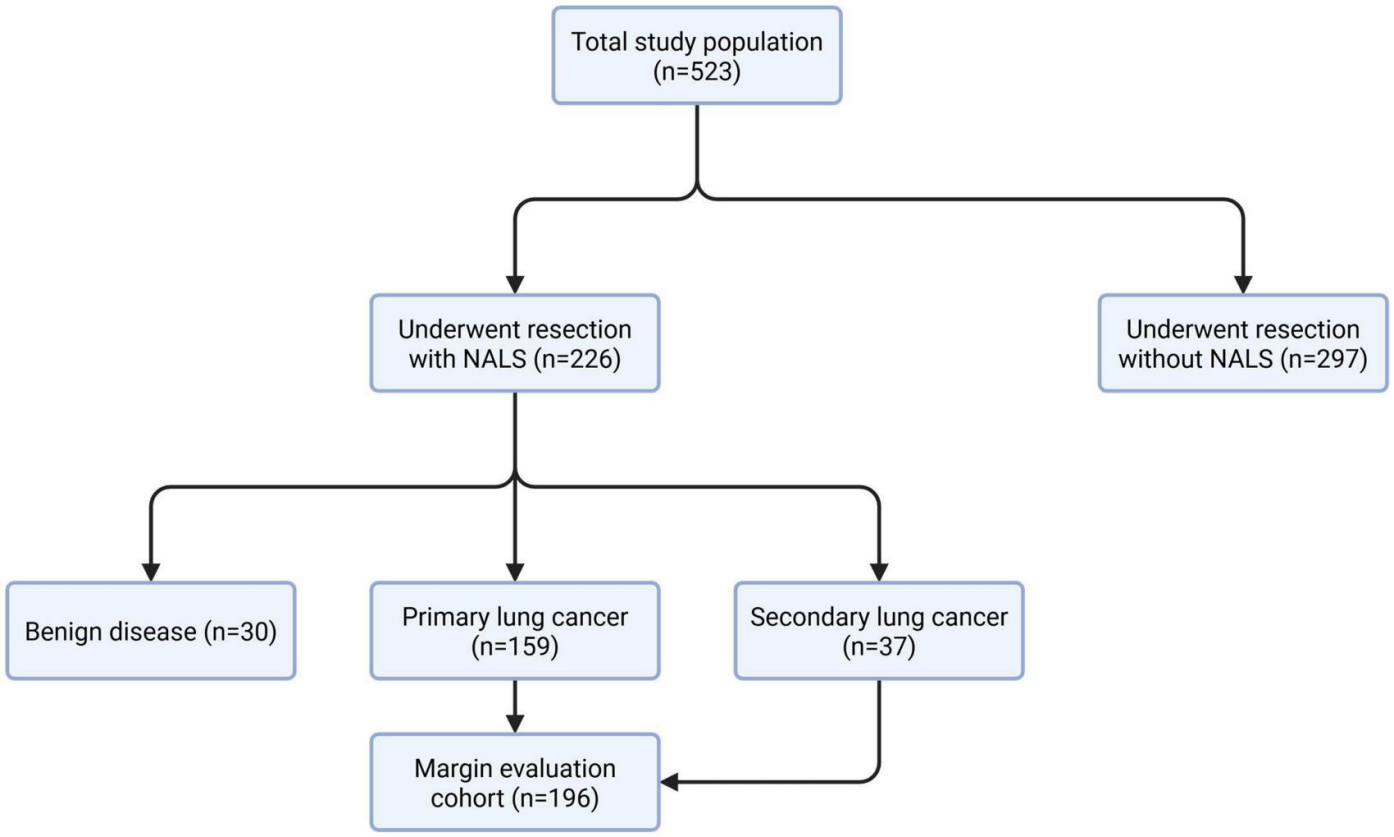
Flow Diagram of the Study Population. Of 523 patients who underwent pulmonary resection during the study period, 226 underwent resection using the novel asymmetrical linear stapler (NALS). Margin evaluation was performed in those with malignant final pathology. Figure 1 was created by the authors using BioRender.com under an appropriate publication license.

A retrospective medical record review was conducted for patients who underwent pulmonary resection at our institution between July 2024 and December 2025. Patients who underwent pulmonary resection using the NALS for lung masses requiring surgical excision because of suspected or confirmed malignancy were included. The NALS was applied to parenchymal or bronchial resection margins according to the surgical scenario (**Figure 1**). This study was approved by the Institutional Review Board of Chungnam National University Hospital (CNUH 2025–08-075), and the requirement for informed consent was waived because of the retrospective study design.

All procedures were performed using VATS under general anaesthesia with double-lumen endotracheal intubation. In selected cases, electromagnetic navigation bronchoscopy (ENB) was utilized intraoperatively to localize and mark the target lesion before resection.^29^^,^^30^ For segmentectomy, intersegmental planes were identified after division of the segmental vessels using intravenous indocyanine green (ICG) and near-infrared (NIR) fluorescence thoracoscopy.^31^ All operations were performed by 2 thoracic surgeons. The extent of pulmonary resection was determined based on tumour characteristics, radiologic suspicion of malignancy, and patient condition. Sublobar resection, including wedge resection or segmentectomy, was selected for peripheral tumours 2 cm or smaller and for patients considered high risk for lobectomy. A lobectomy was performed for larger or centrally located tumours.

### NALS device characteristics and cartridge selection

**Figure 2.**
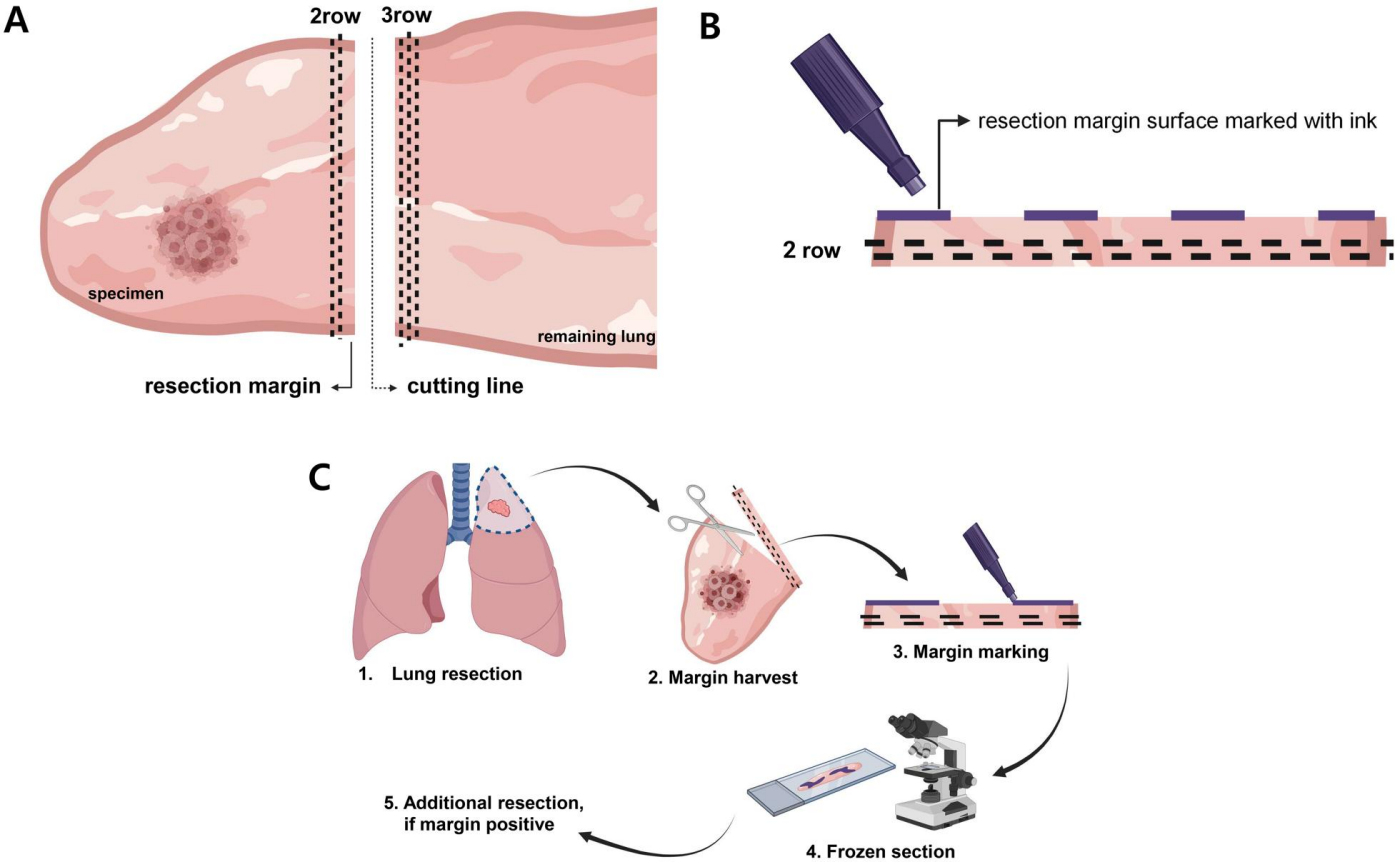
Structural Concept and Integrated Workflow of the Novel Asymmetric Linear Stapler (NALS). (A) Pulmonary parenchymal division using the NALS, with triple-row staples on the remaining lung side and double-row staples on the specimen side. (B) Specimen orientation with inking of the outer resection margin. (C) Integrated workflow for intraoperative frozen-section examination. Figure 2 was created by the authors using BioRender.com under an appropriate publication license.

The NALS incorporates an asymmetrical staple-line configuration with a double row of staples on the specimen side and a triple row on the remaining lung side, with an integrated cutter between them. This design allows direct access to the anatomical resection margin on the specimen side for intraoperative margin assessment (**Figure 2**). Two cartridge types were used: green (open height: 4.5 mm, closed height: 2.0 mm) for lobar bronchi and thick parenchyma, and blue (open height: 3.5 mm, closed height: 1.5 mm) for segmental bronchi and thinner parenchyma. Cartridge selection was made intraoperatively at the surgeon’s discretion.

### Intraoperative frozen-section and margin assessment workflow

In patients without a definitive preoperative diagnosis, an intraoperative frozen section was performed to confirm the nature of the primary lesion. In patients with malignancy confirmed preoperatively or intraoperatively, additional frozen-section examination was performed for the parenchymal or bronchial resection margin according to the procedure.

After resection, the surgeon harvested a specimen including the double-row staple line adjacent to the anatomical resection margin for frozen-section analysis. The outer margin was marked with surgical ink before transport to pathology. The specimen was oriented with the staple line inferiorly and the inked outer margin superiorly during freezing. Pathological assessment was performed by an experienced lung pathologist. Because frozen-section interpretation was part of intraoperative clinical decision-making, formal blinding to the surgical context was not applied. The integrated surgical-pathological workflow is shown in **Figure 2**.

### Study endpoints

The primary objective of this study was to evaluate the feasibility of intraoperative resection margin assessment using the NALS. Margin status was assessed in cases with malignant final pathology.

## RESULTS

### Patient cohort and baseline characteristics

During the study period, 523 patients underwent pulmonary resection at our institution. Of these, 226 (43.2%) underwent pulmonary resection using the NALS and were included in this study. Baseline demographic, radiologic, and surgical characteristics are summarized in **Table 1**. Descriptive characteristics of patients who did and did not undergo NALS use are provided in **Table S1**.

**Table 1.** Baseline Patient and Surgical Characteristics (*n* = 226)

Variable	Value
**Patient characteristics**	
Age, years, mean ± SD (range)	66.9 ± 9.1 (30-83)
Male sex, *n* (%)	124 (54.9)
Female sex, *n* (%)	102 (45.1)
**Final pathological diagnosis, *n* (%)**	
Benign disease	30 (13.3)
Primary lung cancer	159 (70.4)
Secondary lung cancer	37 (16.4)
**Surgical characteristics**	
**Type of pulmonary resection, *n* (%)**	
Wedge resection	124 (54.9)
Segmentectomy	48 (21.2)
Lobectomy	54 (23.9)
**Adjunct techniques, *n* (%)**	
Electromagnetic navigation bronchoscopy	71 (31.4)
Indocyanine green fluorescence^a^	40 (83.3)^b^
**Tumour characteristics (malignant cases, *n* = 196)**	
Tumour size on preoperative CT, mm, median (range)	17 (5-63)
**Resection margin evaluation** ^c^	
Parenchymal resection margin evaluated, *n* (%)	144 (83.7)^d^
Bronchial resection margin evaluated, *n* (%)	62 (60.8)^e^

a Indocyanine green fluorescence was used for intraoperative identification of the intersegmental plane during segmentectomy.

b Percentage calculated among patients who underwent segmentectomy (*n* = 48).

c Margin counts represent the number of margins evaluated and are not mutually exclusive, as some procedures (eg, segmentectomy) involved evaluation of both parenchymal and bronchial margins.

d Percentage calculated among patients who underwent wedge resection or segmentectomy (*n* = 172).

e Percentage calculated among patients who underwent segmentectomy or lobectomy (*n* = 102).

The mean age was 66.9 ± 9.1 years (median, 68.0 years), and 124 patients (54.9%) were male. Wedge resection was performed in 124 patients, segmentectomy in 48, and lobectomy in 54. Electromagnetic navigation bronchoscopy-guided lung marking was performed in 71 cases, and ICG was used for intersegmental plane identification in 40 segmentectomies. Final pathology showed benign disease in 30 patients (13.3%), primary lung cancer in 159 (70.4%), and secondary lung cancer in 37 (16.4%).

### Resection margin status

**Figure 3.**
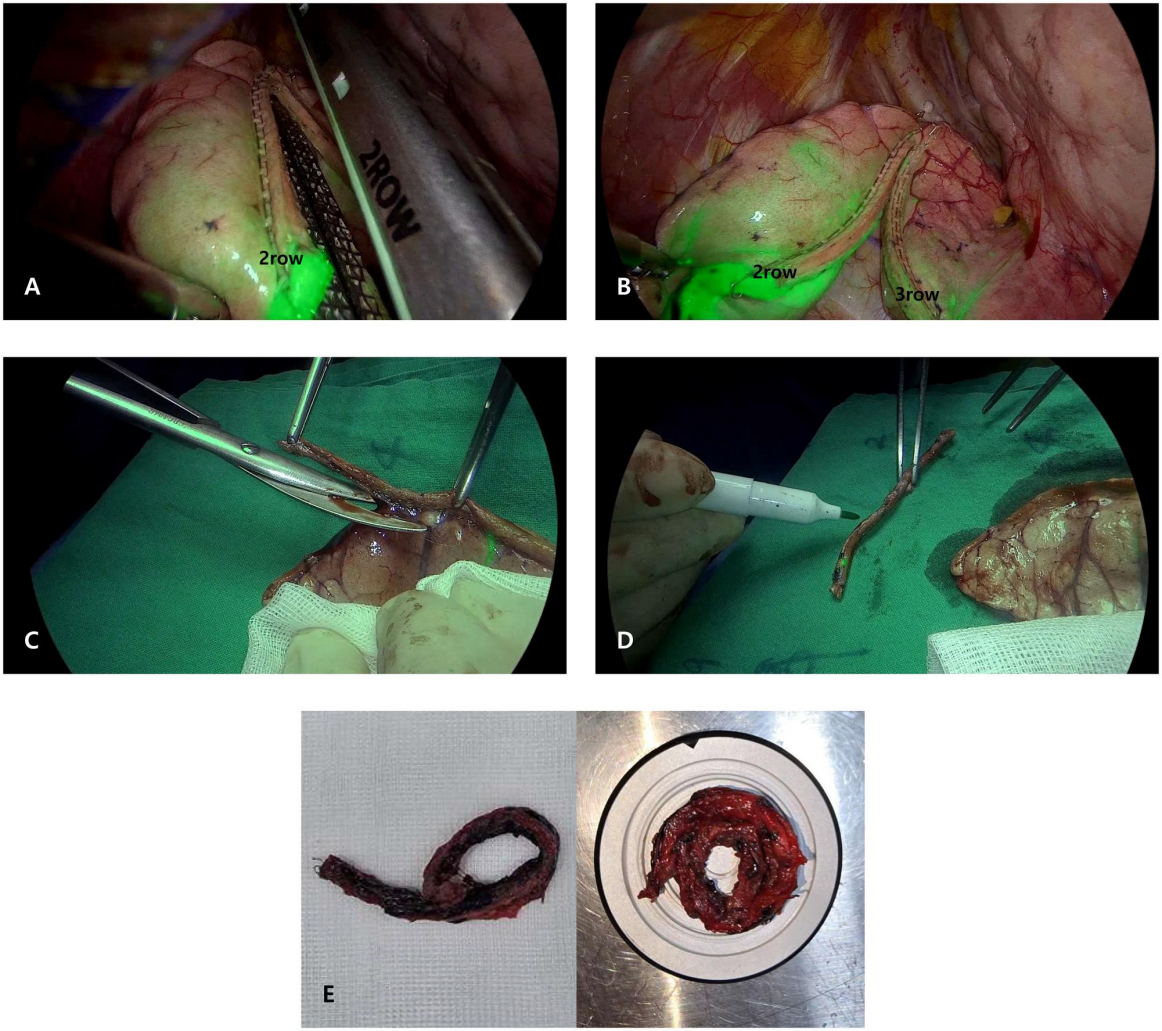
Intraoperative Workflow for Resection Margin Assessment Using the NALS. (A) Application of the NALS during pulmonary parenchymal division. (B) Resected specimen and remaining lung after division, with double-row staples on the specimen side and triple-row staples on the remaining lung side. (C) Harvest of the resection margin specimen. (D) Marking of the outer resection margin. (E) Frozen-section processing with the inked outer margin oriented superiorly and the staple line inferiorly.

Resection margin assessment was performed on a margin-by-margin basis. A total of 206 margins were evaluated, including 144 parenchymal and 62 bronchial margins. Intraoperative frozen-section examination was successfully performed in all evaluated margins. Representative specimen handling and frozen-section preparation are shown in **Figure 3**.

Overall, 12 margins (5.8%) were categorized as positive on intraoperative frozen-section examination. Parenchymal margin positivity was identified in 8 of 144 margins (5.6%), and bronchial margin positivity in 4 of 62 margins (6.5%) (**Table 2**).

**Table 2.** Intraoperative Resection Margin Assessment Using NALS^d^

Variable	Parenchymal margins	Bronchial margins
	(*n* = 144)	(*n* = 62)
**Margin positivity by margin type**		
Positive margins, *n* (%)	8 (5.6)	4 (6.5)
**Resection margin distance in frozen-section-negative margins**		
Measurement available, *n* (%)^a^^,^^b^	108 (79.4)	58 (100)
Median (range), mm	10 (1-40)	20 (2-73)
Margins ≥10 mm, *n* (%)	72 (66.7)	49 (84.5)
Margins ≥20 mm, *n* (%)	20 (18.5)	28 (48.3)
**Correlation between intraoperative frozen-section and permanent pathology**		
Frozen section positive, *n* (%)	8 (5.6)	4 (6.5)
Permanent positive, *n* (%)	4 (2.8)	0 (0)
Permanent negative, *n* (%)	4 (2.8)	4 (6.5)
Frozen section negative, *n* (%)	136 (94.4)	58 (6.5)
Permanent positive, *n* (%)	0 (0)	0 (0)
Permanent negative, *n* (%)	136 (94.4)	58 (93.5)
**Exploratory diagnostic performance of intraoperative frozen section** ^c^		
Sensitivity, %	100	Not estimable
Specificity, %	97.1	93.5
Positive predictive value, %	50.0	0
Negative predictive value, %	100	100

a Percentage calculated among frozen-section-negative parenchymal margins (*n* = 136).

b Percentage calculated among frozen-section-negative bronchial margins (*n* = 58).

c Diagnostic performance metrics were calculated on a margin-by-margin basis using permanent pathology as the reference standard. These values should be interpreted cautiously because of the very small number of permanent margin-positive cases, particularly for bronchial margins, in which no permanent-positive cases were observed.

^d^Resection margin assessment was performed on a margin-by-margin basis; parenchymal and bronchial margins could overlap in the same patient, particularly in segmentectomy cases.

### Resection margin distance

Margin distance analysis was performed only for margins reported as negative on the intraoperative frozen-section examination and with available measurements. Among 136 negative parenchymal margins, measurements were available in 108 cases. The median parenchymal margin distance was 10 mm (range, 1-40 mm); 72 margins (66.7%) were at least 10 mm, including 20 (18.5%) that were at least 20 mm (**Table 2**).

Among 58 negative bronchial margins, measurements were available in all cases. The median bronchial margin distance was 20 mm (range, 2-73 mm); 49 margins (84.5%) were at least 10 mm, including 28 (48.3%) that were at least 20 mm (**Table 2**).

### Correlation between intraoperative frozen-section and permanent pathology

Correlation between intraoperative frozen section findings and permanent pathological results differed by margin type (**Table 2**). Among the 8 parenchymal margins categorized as positive frozen-section, 2 demonstrated definite tumour involvement, whereas the remainder showed indeterminate findings, including atypical pneumocystic proliferation, atypical acinar structures, mucinous material with atypical cells, or a solid lesion of undetermined significance. Residual tumour was confirmed on permanent pathology in 4 margins, whereas the remaining 4 margins demonstrated reactive or indeterminate changes without residual tumours.

**Figure 4.**
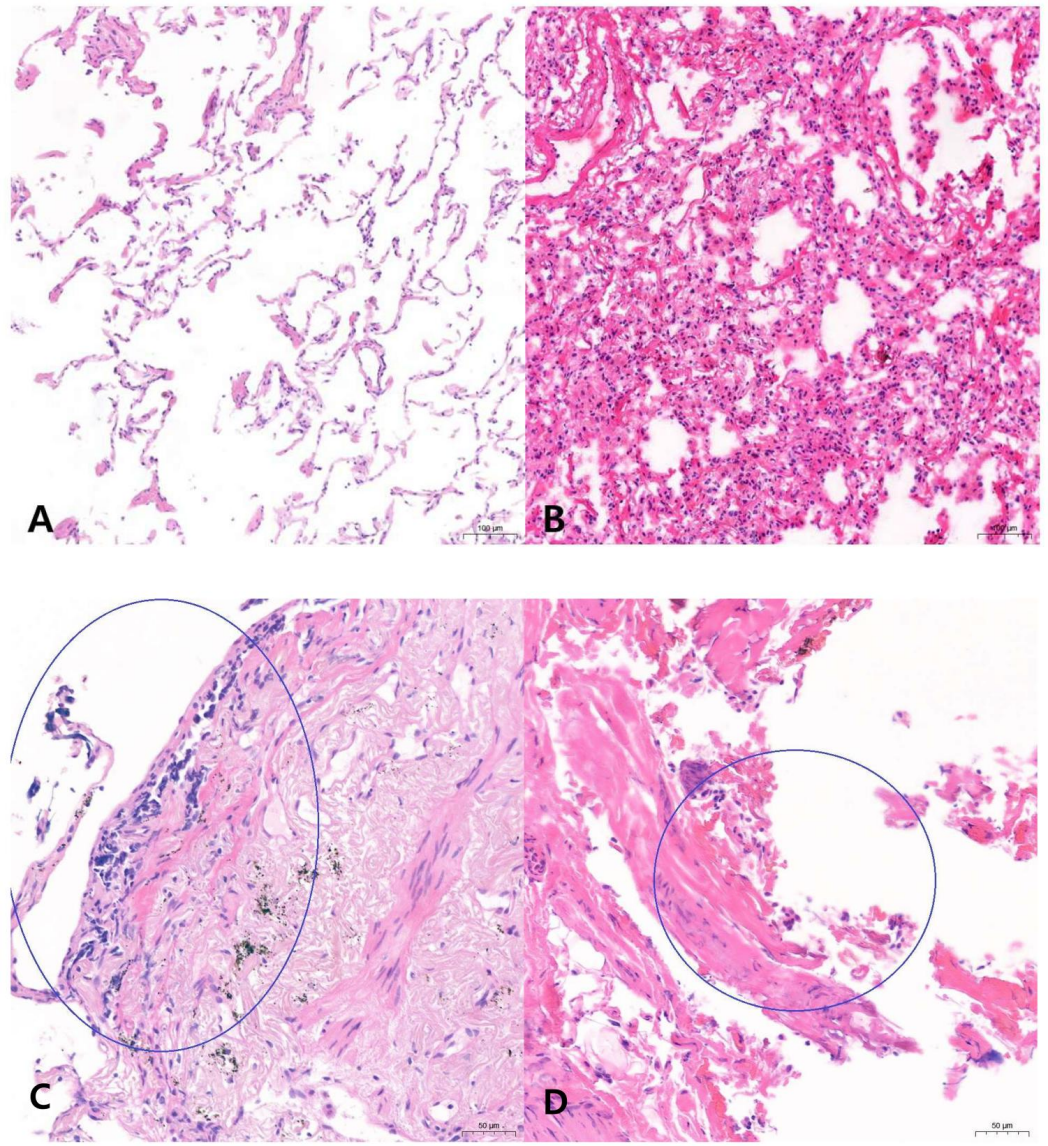
Representative Histopathologic Findings of Resection Margins Assessed Using the Novel Asymmetric Linear Stapler (NALS). (A) Negative parenchymal resection margin (HPF ×100). (B) Negative bronchial resection margin (HPF ×100). (C) Positive parenchymal resection margin with tumour infiltration at the true resection margin, indicated by the circled area (HPF ×200). (D) Bronchial resection margin of case 30, showing atypical epithelial cells on frozen section, indicated by the circled area, without residual tumour on permanent pathology (HPF ×200).

All 4 bronchial margins categorized as positive on intraoperative frozen-section examination demonstrated indeterminate or reactive epithelial changes rather than definite tumour involvement. On permanent pathology, none showed a residual tumour; the findings were consistent with squamous metaplasia or reactive epithelial hyperplasia. Representative histopathologic findings of the resection margin assessed using NALS are shown in **Figure 4**. No margin reported as negative on intraoperative frozen-section examination was found to be positive on permanent pathology.

Using permanent pathology as the reference standard, exploratory margin-level diagnostic performance of intraoperative frozen-section assessment was calculated. For parenchymal margins, sensitivity was 100% (4/4), specificity 97.1% (136/140), positive predictive value 50.0% (4/8), and negative predictive value 100% (136/136). For bronchial margins, specificity was 93.5% (58/62), positive predictive value 0%, and negative predictive value 100%; sensitivity could not be estimated because no bronchial margins were positive on permanent pathology. Overall pooled sensitivity was 100% (4/4), specificity 96.0% (194/202), positive predictive value 33.3% (4/12), and negative predictive value 100% (194/194). These values should be interpreted cautiously given the very small number of permanent margin-positive cases.

### Intraoperative management of margin-positive cases

In margin-positive cases, intraoperative frozen-section findings directly influenced surgical decision-making. Additional parenchymal or bronchial resection was performed when feasible, whereas alternative strategies, including margin marking or adjuvant therapy, were selected when further anatomical resection was not possible.

## DISCUSSION

### Principal findings

In this initial single-institution experience, intraoperative resection margin assessment using the NALS was feasible and enabled structured evaluation of the anatomical resection margin during pulmonary resection. No margin reported as negative on intraoperative frozen-section examination was identified as positive on permanent pathological evaluation in this cohort. Margin positivity was identified intraoperatively in 5.8% of evaluated margins, supporting the clinical relevance of real-time margin assessment during pulmonary resection.^10^^,^^13^ The principal benefit of the NALS is not proven oncologic superiority over conventional staplers, but its asymmetric staple-line configuration, which enables direct access to the anatomical resection margin on the specimen side for intraoperative pathological examination. In contrast, conventional staplers generally produce a symmetric, mechanically compressed staple line that limits direct assessment of the actual resection plane.^9^^,^^26^^,^^28^ Accordingly, the present study should be interpreted as a feasibility evaluation of anatomical margin accessibility and pathology workflow integration rather than as evidence of comparative oncologic superiority.

### The current problem in margin assessment

Accurate margin evaluation after pulmonary resection remains challenging when conventional staplers are used. Mechanical compression and crush injury may distort the resection surface and complicate interpretation of the anatomical resection margin.^6^^,^^8^ In addition, staples may interfere with histologic processing and may require manual removal, potentially causing further tissue disruption.^8^^,^^14^^,^^32^ Prior studies have emphasized the importance of intraoperative margin verification during sublobar resection, but conventional workflows have generally relied on indirect approaches such as surgical margin cytology or frozen-section pathology rather than direct harvesting of the stapled anatomical resection margin.^12^^,^^24^^,^^33^ These methodological constraints contribute to ongoing debate regarding optimal margin thresholds in lung cancer surgery.^34^^,^^35^ Preclinical comparative data for the NALS also suggested that conventional staplers may cause marked squeezing artefacts at the stapled margin, making direct pathological evaluation difficult.^9^ Taken together, these findings suggest that the unmet need lies not in recognizing the importance of margin assessment itself, but in improving access to the actual stapled resection plane. The present study should therefore be interpreted as an initial clinical feasibility evaluation of a device designed to address that technical limitation.

### Margin distance versus true margin status

Traditional oncologic principles have emphasized numerical margin distance, often using fixed thresholds such as a 2-cm or a margin equal to tumour size. However, such measurements are usually derived from permanent pathological specimens and may not accurately reflect the intraoperative resection plane.^11^^,^^12^^,^^15^^,^^32^ In the present study, margin assessment was performed directly at the anatomical resection surface. Although sublobar resections predominated and parenchymal margin distances were correspondingly short, no false-negative frozen-section findings were identified in this series. Given the limited sample size, however, this observation should be interpreted cautiously and does not permit definitive conclusions regarding diagnostic performance.

### Pathologic difference between parenchymal and bronchial margins

Distinct patterns were observed between parenchymal and bronchial resection margins. Among parenchymal margins categorized as positive on frozen-section examination, findings ranged from definite tumour involvement to indeterminate atypical epithelial changes, and residual tumour was confirmed on permanent pathology in 4 of 8 cases. In contrast, all bronchial margins categorized as positive on intraoperative frozen-section examination were negative on permanent pathological evaluation and demonstrated only reactive or metaplastic epithelial changes. These findings suggest that bronchial frozen-section interpretation may be more prone to overcalling in the intraoperative setting, because reactive hyperplasia and metaplastic epithelial changes can mimic malignancy in limited frozen-section material. Accordingly, bronchial frozen-section and permanent-pathology discordance in the present cohort should be interpreted cautiously as a potential interpretive limitation of intraoperative bronchial margin assessment rather than as definitive evidence of residual tumour.^8^^,^^32^

### Surgical impact of frozen-permanent discordance

Among the 12 margins categorized as positive on intraoperative frozen-section examination, residual tumour was confirmed on permanent pathology in 4 parenchymal margins. The remaining 4 parenchymal margins and all 4 bronchial margins demonstrated discordant frozen-section and permanent-pathology findings. Importantly, the surgical implications of these discordant findings were limited. All bronchial margin–positive cases occurred during lobectomy, and additional bronchial resection using the NALS was performed without altering the planned anatomical extent of resection. Similarly, all parenchymal margin–positive cases occurred during sublobar resection, and additional resection was achieved by extending the stapler line along the existing parenchymal margin without conversion to lobectomy. Thus, although frozen-section overestimation occurred in a subset of cases, additional resections remained within the originally intended anatomical framework. In contrast, no false-negative frozen-section findings were identified in this cohort, suggesting that intraoperative assessment favoured oncologic caution while preserving the overall surgical strategy.

### Implication for parenchyma-sparing surgery

Randomized trials such as JCOG0802 and CALGB 140503 have demonstrated comparable survival outcomes between sublobar resection and lobectomy in selected early-stage tumours, although differences in local recurrence have been reported.^21^^,^^22^ Reliable intraoperative margin assessment may therefore be particularly relevant in the context of parenchyma-sparing surgery. In clinical practice, this potential advantage may be greatest during sublobar resections, where uncertainty regarding margin adequacy can directly influence the extent of resection. By enabling pathological assessment at the anatomical resection plane, the NALS may provide additional intraoperative information when surgeons must balance oncologic caution against preservation of functional lung parenchyma, particularly in patients with limited physiological reserve or small peripheral tumours.^25^ Its potential clinical contribution therefore lies in supporting more informed margin assessment during parenchyma-sparing resection rather than simply promoting wider resection. In margin-positive cases, frozen-section findings directly influenced intraoperative decision-making, and additional parenchymal or bronchial resection was performed when feasible. These observations suggest that the NALS may function not merely as a stapling device but as a platform for real-time, pathology-guided intraoperative decision-making.

### Limitations

This study has several limitations. First, it represents a single-institution experience without a control group using conventional staplers, which precludes direct comparison and prevents conclusions regarding superiority of the NALS over standard stapling devices. Second, pathological assessment was performed by a single experienced pathologist without formal blinding to the surgical context, which may limit generalizability and introduce observer-related bias. Third, the number of permanent margin-positive cases was small, limiting robust estimates of diagnostic performance, particularly for bronchial margins, in which no permanent-positive cases were identified. Fourth, objective quantitative assessment of staple-related tissue artefact was not performed; histologic interpretability was evaluated descriptively, and the present study, therefore, does not provide formal evidence that the NALS improves tissue quality for pathological analysis compared with conventional staplers. Finally, long-term oncologic outcomes, including local recurrence, disease-free survival, and overall survival, were not assessed. Accordingly, the present findings should be interpreted as a feasibility evaluation of intraoperative anatomical margin assessment and workflow integration rather than as evidence of comparative clinical benefit, oncologic superiority, or improved histologic assessment.

## CONCLUSIONS

In conclusion, intraoperative resection margin assessment using the NALS was feasible and enabled systematic evaluation of the anatomical resection margin during pulmonary resection. No false-negative frozen-section findings were identified in this series, although the limited number of permanent margin-positive cases precludes definitive conclusions regarding diagnostic performance. Because no control group using conventional staplers was included, the present study should be interpreted as a feasibility assessment of pathology-guided intraoperative margin evaluation rather than as evidence of superiority over standard stapling devices. Further multicentre studies incorporating controlled comparisons and long-term oncologic follow-up are warranted.

## Supplementary Material

ivag140_Supplementary_Data

## Data Availability

Data are available from the corresponding author on reasonable request, subject to institutional and ethical restrictions.

## References

[1] Lee GD , Kim DK, Jang SJ, et al Significance of R1-resection at the bronchial margin after surgery for non-small-cell lung cancer. Eur J Cardiothorac Surg. 2017;51:176-181. 10.1093/ejcts/ezw242 27401705

[2] Rami-Porta R , Wittekind C, Goldstraw P. Complete resection in lung cancer surgery: from definition to validation and beyond. J Thorac Oncol. 2020;15:1815-1818. 10.1016/j.jtho.2020.09.006 33067147

[3] Rami-Porta R. The evolving concept of complete resection in lung cancer surgery. Cancers (Basel). 2021;13:2583. 34070418 10.3390/cancers13112583 PMC8197519

[4] Kaiser LR. Video-assisted thoracic surgery. Current state of the art. Ann Surg. 1994;220:720-734. 10.1097/00000658-199412000-00003 7986137 PMC1234472

[5] Sihoe AD. Video‐assisted thoracoscopic surgery as the gold standard for lung cancer surgery. Respirology. 2020;25:49-60. 10.1111/resp.13920 32734596

[6] Burgio V , Bei J, Rodriguez Reinoso M, et al Mechanical stapling devices for soft tissue repair: a review of commercially available linear, linear cutting, and circular staplers. Appl Sci. 2024;14:2486. 10.3390/app14062486

[7] Tsujimoto H , Tsuda H, Hiraki S, et al In vivo evaluation of a modified linear stapling device designed to facilitate accurate pathologic examination of the surgical margin. Gastric Cancer. 2016;19:666-669. 10.1007/s10120-015-0520-1 26199024

[8] Borczuk AC. Challenges of frozen section in thoracic pathology: lepidic lesions, limited resections, and margins. Arch Pathol Lab Med. 2017;141:932-939. 10.5858/arpa.2016-0415-ra 27763791

[9] Kang S-K , San Bok J, Cho HJ, Kang M-W. Novel asymmetrical linear stapler (NALS) for pathologic evaluation of true resection margin tissue. J Thorac Dis. 2018;10:S1631-S1636. 30034828 10.21037/jtd.2018.03.158 PMC6035920

[10] Predina JD , Keating J, Patel N, Nims S, Singhal S. Clinical implications of positive margins following non‐small cell lung cancer surgery. J Surg Oncol. 2016;113:264-269. 10.1002/jso.24130 26719121

[11] Wolf AS , Swanson SJ, Yip R, et al; I-ELCAP Investigators. The impact of margins on outcomes after wedge resection for stage I non-small cell lung cancer. Ann Thorac Surg. 2017;104:1171-1178. 28669499 10.1016/j.athoracsur.2017.04.024

[12] Nagano M , Sato M. Impact of surgical margin after sublobar resection of lung cancer: a narrative review. J Thorac Dis. 2023;15:5750. 37969293 10.21037/jtd-23-711 PMC10636452

[13] Wong L-Y , Dale R, Kapula N, et al Impacts of positive margins and surgical extent on outcomes after early-stage lung cancer resection. Ann Thorac Surg. 2024;118:1126-1134. 38866199 10.1016/j.athoracsur.2024.05.032

[14] Batsakis JG. Surgical excision margins: a pathologist’s perspective. Adv Anat Pathol. 1999;6:140-148. 10342011 10.1097/00125480-199905000-00002

[15] Goldstein NS , Ferkowicz M, Kestin L, Chmielewski GW, Welsh RJ. Wedge resection margin distances and residual adenocarcinoma in lobectomy specimens. Am J Clin Pathol. 2003;120:720-724. 10.1309/p47fyw5u4crq0wfe 14608898

[16] Moon Y , Park JK, Lee KY. The effect of resection margin distance and invasive component size on recurrence after sublobar resection in patients with small (≤2 Cm) lung adenocarcinoma. World J Surg. 2020;44:990-997. 10.1007/s00268-019-05276-5 31712844

[17] Gossot D , Lafouasse C, Kovacs E, Seguin-Givelet A. Sublobar resection for early-stage lung cancer: the issue of safety margins. Eur J Cardiothorac Surg. 2023;63:ezad055. 10.1093/ejcts/ezad055 36810786

[18] Kamtam DN , Berry MF, Lui NS, et al What is an adequate margin during sublobar resection of≤ 3 cm N0 subsolid lung adenocarcinomas? Ann Thorac Surg. 2024;118:801-809. 38734402 10.1016/j.athoracsur.2024.04.018 PMC12704906

[19] Huang L , Petersen RH. Impact of margin distance on locoregional recurrence and survival after thoracoscopic segmentectomy. Ann Thorac Surg. 2025;119:316-324. 39067631 10.1016/j.athoracsur.2024.07.012

[20] Suzuki K , Watanabe S, Wakabayashi M, et al A nonrandomized confirmatory phase III study of sublobar surgical resection for peripheral ground glass opacity dominant lung cancer defined with thoracic thin-section computed tomography (JCOG0804/WJOG4507L). J Clin Oncol. 2017;35:8561.

[21] Saji H , Okada M, Tsuboi M, et al; West Japan Oncology Group and Japan Clinical Oncology Group. Segmentectomy versus lobectomy in small-sized peripheral non-small-cell lung cancer (JCOG0802/WJOG4607L): a multicentre, open-label, phase 3, randomised, controlled, non-inferiority trial. Lancet. 2022;399:1607-1617. 35461558 10.1016/S0140-6736(21)02333-3

[22] Altorki N , Wang X, Kozono D, et al Lobar or sublobar resection for peripheral stage IA non–small-cell lung cancer. N Engl J Med. 2023;388:489-498. 36780674 10.1056/NEJMoa2212083 PMC10036605

[23] Aokage K , Suzuki K, Saji H, et al; Japan Clinical Oncology Group. Segmentectomy for ground-glass-dominant lung cancer with a tumour diameter of 3 cm or less including ground-glass opacity (JCOG1211): a multicentre, single-arm, confirmatory, phase 3 trial. Lancet Respir Med. 2023;11:540-549. 36893780 10.1016/S2213-2600(23)00041-3

[24] Ortiz BA , Engrav SK, Roden AC, et al Impact of frozen section pathology examination of surgical margins in sublobar pulmonary resections for clinical stage IA non-small cell lung cancer. Ann Thorac Surg. 2025;120:1044-1051. 40288733 10.1016/j.athoracsur.2025.04.005

[25] Krantz SB , Mitzman B, Antonoff MB, et al; ThORN. Thoracic surgery outcomes research network (ThORN) consensus document on defining a high-quality wedge resection for early-stage lung cancer. Ann Thorac Surg. 2025;119:944-956. 39793687 10.1016/j.athoracsur.2024.12.017

[26] Jung YC , Piao WX, Kim J-y, et al Novel asymmetrical linear stapler: safety test and pathological assessment in a porcine model. J Surg Res. 2024;304:58-66. 39522404 10.1016/j.jss.2024.10.003

[27] Kang MW. Evolution of lung cancer surgery: historical milestones, current strategy, and future innovations. J Chest Surg. 2025;58:79-84. 10.5090/jcs.25.025 40230346 PMC12066400

[28] Chong Y , Kang M-W. Initial experience and feasibility of novel asymmetric linear stapler in pulmonary resection: single center experience. J Chest Surg. 2025;58:S86-S88.

[29] Cho HJ , Roknuggaman M, Han WS, Kang SK, Kang M-W. Electromagnetic navigation bronchoscopy—Chungnam National University Hospital experience. J Thorac Dis. 2018;10:S717-S724. 10.21037/jtd.2018.03.130 29732192 PMC5911745

[30] Piao Z , Han SJ, Cho HJ, Kang M-W. Feasibility of electromagnetic navigation bronchoscopy-guided lung resection for pulmonary ground-glass opacity nodules. J Thorac Dis. 2020;12:2467-2473. 32642153 10.21037/jtd.2020.03.71 PMC7330407

[31] Mun M. Intersegmental Plane: Indocyanine Green. Video-Atlas of VATS Pulmonary Sublobar Resections. Springer; 2023:39-43.

[32] Han Y , Cai G. Intraoperative frozen section diagnosis of lung specimens: An updated review. Elsevier; 2025:150901. 10.1016/j.semdp.2025.150901 40188626

[33] Sawabata N , Karube Y, Umezu H, et al Cytologically malignant margin without continuous pulmonary tumor lesion: cases of wedge resection, segmentectomy and lobectomy. Interact CardioVasc Thorac Surg. 2008;7:1044-1048. 10.1510/icvts.2008.184192 18782788

[34] Edwards JG , Chansky K, Van Schil P, et al; International Association for the Study of Lung Cancer Staging and Prognostic Factors Committee, Advisory Board Members, and Participating Institutions. The IASLC lung cancer staging project: analysis of resection margin status and proposals for residual tumor descriptors for non–small cell lung cancer. J Thorac Oncol. 2020;15:344-359. 31731014 10.1016/j.jtho.2019.10.019

[35] Detterbeck FC , Ostrowski M, Hoffmann H, et al; Jul Members of the Staging and Prognostic Factors Committee and Advisory Boards. The international association for the study of lung cancer lung cancer staging project: proposals for revision of the classification of residual tumor after resection for the forthcoming (ninth) edition of the TNM classification of lung cancer. J Thorac Oncol. 2024;19:1052-1072. 10.1016/j.jtho.2024.03.021 38569931

