# Supplementary material for: Intraoperative Assessment of Anatomical Resection Margin Using a Novel Asymmetric Linear Stapler: An Initial Single Institution Experience
Source: Interdiscip Cardiovasc Thorac Surg. 2026 May 9;41(6):ivag140. doi: 10.1093/icvts/ivag140 (PMC13250331; doi:10.1093/icvts/ivag140)
Supplement: ivag140_Supplementary_Data [file ivag140_supplementary_data.docx]

**Supplementary Table 1. Baseline characteristics of patients who underwent pulmonary resection during the study period according to NALS use**

| Variable | NALS group  (n = 226) | Non-NALS group  (n = 297) |
| --- | --- | --- |
| Age, y (Mean ± SD) | 66.9 ± 9.1 | 63.7 ± 8.7 |
| Sex  Male, n (%)  Female, n (%) | 124 (54.9)  102 (45.1) | 152 (51.2)  145 (48.8) |
| Final diagnosis, n (%)  Benign disease  Primary lung cancer  Secondary lung cancer | 30 (13.3)  159 (70.4)  37 (16.4) | 36 (12.1)  205 (69.0)  56 (18.9) |
| Surgical approach, n (%)  Thoracotomy  VATS  Robotic | 0 (0)  226 (100)  0 (0) | 6 (2.0)  278 (93.6)  13 (4.4) |
| Extent of resection, n (%)  Wedge resection  Segmentectomy  Lobectomy | 124 (54.9)  48 (21.2)  54 (23.9) | 104 (35.0)  65 (21.9)  128 (43.1) |

NALS, novel asymmetric linear stapler; VATS, video-assisted thoracic surgery.

**Footnote:** Data are presented descriptively to illustrate selection patterns for NALS use during the study period and should not be interpreted as a formal comparative efficacy analysis.
